# Brain+ AlcoRecover: A Randomized Controlled Pilot-Study and Feasibility Study of Multiple-Domain Cognitive Training Using a Serious Gaming App for Treating Alcohol Use Disorders

**DOI:** 10.3389/fpsyt.2021.727001

**Published:** 2021-10-01

**Authors:** Nicolaj Mistarz, Anette Søgaard Nielsen, Kjeld Andersen, Anneke E. Goudriaan, Lotte Skøt, Kim Mathiasen, Tanja Maria Michel, Angelina Isabella Mellentin

**Affiliations:** ^1^Unit for Psychiatric Research, Department of Clinical Research, University of Southern Denmark, Odense, Denmark; ^2^Brain Research-Inter-Disciplinary Guided Excellence, Department of Clinical Research, University of Southern Denmark, Odense, Denmark; ^3^Amsterdam University Medical Centers, Department of Psychiatry, University of Amsterdam, Amsterdam, Netherlands; ^4^Department of Research, Amsterdam Institute for Addiction Research, Arkin, Amsterdam, Netherlands; ^5^Centre for Telepsychiatry, Mental Health Services of Southern Denmark, Odense, Denmark

**Keywords:** alcohol use disorder, cognitive disability, cognitive training, randomized controlled trial, feasibility

## Abstract

**Background:** Patients with alcohol use disorder (AUD) exhibit deficits in various cognitive domains, including executive functioning, working memory, and learning and memory, which impede the effectiveness of conventional AUD treatment and enhance relapse. Mobile health (mHealth) services are promising in terms of delivering cognitive training in gamified versions. So far, studies examining the effects of mHealth-based cognitive training in AUD patients have, however, focused on specific rather than multiple cognitive domains and overlooked the importance of clinical outcomes. Furthermore, research has yet to investigate the acceptability and feasibility of this type of cognitive training.

**Aims:** The aims of this pilot study are to examine (1) whether using smartphone-based, multi-domain cognitive training with gamified elements as part of conventional treatment for AUD indicate effect, and (2) whether the intervention is acceptable and feasible as a part of conventional treatment for AUD.

**Methods:** Patients from the alcohol outpatient clinic, Odense Municipality, Denmark will be invited to participate in the study on a consecutive basis until a total of 60 patients have been recruited. The study will be performed as a combined parallel randomized controlled trial (RCT) and qualitative feasibility study. The patients will be randomly assigned to one of two groups. The intervention group (*n* = 30) will receive smartphone-based, multi-domain cognitive training with gamified elements together with treatment as usual (TAU). The active control group (*n* = 30) will receive a sham version of the same cognitive training together with TAU. Cognitive outcomes will be assessed *via* the training application at baseline and post-treatment. Clinical outcomes will be assessed at baseline, post-treatment, and at 6-month follow-up using the Addiction Severity Index. Furthermore, the 30 patients randomized to the intervention group will be invited to participate in the second phase, that is the feasibility study, at post-treatment. A questionnaire inquiring about the use of mHealth treatment in general will be administered. Further, feedback regarding functionality and meaningfulness of the application in addition to other qualitative aspects relating to the use of the application will be collected. The patients will also be asked to provide suggestions about how to improve and potentially implement the tool.

**Implications:** It is anticipated that this pilot study will provide tentative evidence for the effectiveness of smartphone-based, multi-domain cognitive training as well as information about the usability and feasibility of this type of training, including acceptability and compliance. The study will also contribute with feedback derived from the patients about how to improve and implement the tool. If promising, the findings will be used to plan a large-scale RCT. Since cognitive deficits are not addressed in current treatments for AUD, gamified cognitive training delivered through smartphones may increase the effectiveness of current treatment for AUD as well as introduce more mHealth-based treatment that is both accessible and cost-effective.

## Background

The encumbering nature of alcohol use disorder (AUD) is indisputable—high rates of prevalence, comorbidity with other disorders, and recurrence (1–5) indicate the urgency of efficacious treatment for relapse prevention. Current AUD treatment encompasses psychotherapy and pharmacotherapy, which aim to reduce craving and consumption of alcohol to achieve controlled drinking or abstinence and maintain it (6, 7). However, patients with AUD often have cognitive dysfunctions, and while contemporary evidence-based treatments, such as cognitive behavioral therapy (CBT), may indirectly increase cognitive capacity through the acquirement of alcohol relevant coping skills (6, 8), there is evidence that cognitive deficits tend to persevere and still be present not only at post-treatment but also even after successful treatment with a full year of abstinence [for more information, see (9–11)]. Thus, it seems that current treatments are not adequate to ameliorate cognitive deficits.

The cognitive dysfunctions in AUD involve the domains for processing speed, executive functioning, working memory, and memory (i.e., visual, and verbal memory), underlining that the whole brain is affected by the neurotoxic effects of alcohol (10, 12, 13). Further, cognitive deficits have been found to be associated with worse treatment outcomes and a higher risk of relapse (8), and, therefore, addressing cognitive deficits during treatment for AUD may contribute positively to the long-term outcome (14).

A direct way of targeting cognitive deficits in AUD could be cognitive training. Several studies have investigated the efficacy of cognitive training in subclinical and clinical AUD samples, but these have mostly focused on cognitive training targeting executive functions and working memory [WM; for an overview, see (15)]. In subclinical samples, one study found that training of inhibitory response (i.e., a subdomain of executive functioning) did not lead to improvement on cognitive and clinical drinking outcomes (16), whereas another study training executive functions and WM found an effect on cognitive and clinical drinking outcomes (17). This discrepancy may be explained by the fact that the two studies focused on training different cognitive domains (i.e., inhibition vs. WM). The improvement in cognitive outcomes reported by Houben et al. (17) may also be due to the training tasks being like the cognitive tests used for assessment.

In clinical samples, several studies have demonstrated improvements in cognitive outcomes after cognitive training [for reviews, see (8, 15)]. Overall, the results of cognitive training seem more promising for patients diagnosed with AUD compared to subclinical samples. However, like the subclinical studies, most of the clinical studies performed so far only focused on examining the effects of cognitive training on executive functions and WM [e.g., (18–21)], and only a few recent studies have examined the effects of training multiple cognitive domains [e.g., (22, 23)].

The premise of cognitive training is that the trained cognitive domain(s) can be transferred to neuropsychological tests targeting a similar domain(s) (i.e., proximal transfer) and/or a dissimilar cognitive domain(s) [i.e., distal transfer; (15)]. Most of the studies examining the effects of WM-specific cognitive training have only found proximal transfer effects [e.g., (19–21)], and only one study showed that WM-specific training could be transferred distally to untrained measures of verbal learning and memory (18). Since recent research suggests that patients with AUD present with diffuse and non-specific cognitive deficits [for reviews, see (10, 11)] it is highly relevant to uncover distal transfer effects as well as the effects of multi-domain cognitive training.

To date, little is known about the effects of cognitive training on clinical outcomes (i.e., craving and severity of relapse). Only one clinical study has examined the effects of WM-specific cognitive training on alcohol consumption, which found no effect of the training (21). However, the training was not delivered as adjunctive treatment and the patients completed the training at home without further support, and the findings indicated that cognitive training as stand-alone treatment is unlikely to have a clinically meaningful effect among AUD patients. Rather, it might be effective as an add-on intervention in combination with treatment as usual. Using it as add-on treatment would also allow clinicians, therapists, and other health care providers to become gradually accustomed to the cognitive training as a part of the conventional intervention programs applied at the treatment facilities.

Current options for delivering cognitive training are based on standardized neuropsychological tasks with poor ecological validity (23). It has been reported that patients find it challenging to maintain their attention and motivation during the training sessions, and performing the training requires a solid alliance between the patient and health provider (23, 24). Technological advancements such as electronic health (eHealth) and mobile health (mHealth) services have, therefore, caught interest. They offer innovative approaches for delivering serious gaming over the internet or smartphone devices (25). The term serious gaming (SG) is derived from the notion of gamifying mundane tasks, which refers to the use of game elements in non-gaming contexts (26). Thus, if it is accepted by the patients, this may be a promising method of delivering treatment to AUD patients in a way that is not only effective but also enjoyable during training, which may improve treatment compliance (23, 24). Cognitive specific SG delivered through mHealth services also allows for feedback to be given to the patients, permitting them to track their own progress and accomplishments, which is important for increasing the effectiveness of the training itself (27). Nevertheless, studies examining cognitive specific SG delivered through mHealth services are scarce, and to date, only one study has examined the use of tablets for cognitive training targeting executive functions (23). In this study the authors developed an multimodal application with SG-elements that made use of both visual and auditive stimuli in, which not only resulted in improved executive functioning, but it also showed that mHealth-based training was more motivating and engaging for the patients (23). In addition to the benefits of multimodal treatment delivery, cognitive specific SG delivered through smartphone applications provide the means from which it is possible to create personalized cognitive training programs. This aspect is essential for patients with AUD, which is a heterogenous populations showing diffuse deficits that varies across patients (10, 11). A cognitive training program that constantly adapts to the performance of the patient, would not only be more motivating, but it would also be able to adjust the level of difficulty depending on the qualitative and quantitative pattern of cognitive impairment.

Although a few studies examining cognitive training have applied SG-elements (21, 22), they have, perhaps due to rapid developments in technology, either used less engaging game designs or overlooked the mHealth and eHealth capabilities by constructing games with poor accessibility (i.e., training programs only available for computers). Cognitive specific SG that is either unintuitive or prerequisites specific electronic equipment and demanding hardware, may obstruct the applicability in clinical facilities, which in turn may result in health care providers and patients being less willing to adopt the treatment (27). On the contrary, the results in the study by Gamito et al. (23) highlights that accessible and intuitive mHealth-based treatment with SG-elements delivered on tablets or smartphones may be well-accepted by both patients and therapists. Treatment options that rely on SG-elements have also been shown to be highly feasible and accepted by patients with other mental disorders (28, 29). Nevertheless, current mHealth-based treatments are not proportional to the ongoing technological advancements, which points to the need for more studies examining the feasibility of newer and more modern mHealth-based, multi-domain cognitive training with SG-elements in patients with AUD (i.e., whether the patients will use the training programs as add-on intervention to treatment as usual, and how they will use them). In this process, studies should ensure that patients are involved in developing strategies to use the cognitive training programs as this will help improve and ease the implementation of the intervention.

The aims of this pilot study are to examine (1) whether using smartphone-based, multi-domain cognitive training with gamified elements as part of conventional treatment for AUD shows some effect, and (2) whether the intervention is acceptable and feasible as part of conventional treatment for AUD.

## Methods

### Design

This pilot study will be conducted in two phases: (1) a parallel small-scale randomized controlled trial (RCT) and (2) a feasibility study.

### Setting

The study will be carried out at the outpatient alcohol clinic, Odense Municipality, Denmark. Outpatient treatment is publicly financed and accessible to individuals AUD with varying levels of severity or other alcohol-related problems. Individuals with AUD and comorbid disorders such as psychotic or affective disorders or individuals with other substance-related disorders are presented with treatment options localized at different facilities (30). Furthermore, individuals at the outpatient clinic are offered anonymous treatment.

### Treatment-As-Usual

Before the primary treatment is offered, it is possible for patients to receive a personalized, pharmacological detoxification program at the clinic. Here the patients will be administered the benzodiazepine, chlordiazepoxide, and the specific dosage and duration is adapted to the needs of each individual patient. In 2019, the outpatient facility received 230 patients, where 18.26% (i.e., 42 patients) had undergone pharmacological detoxification.

Psychotherapy and pharmacotherapy are used as the primary treatment either alone or in combination. The former includes motivational interviewing and CBT administered as eight individual or group-based sessions with the option for extension, and the latter often encompasses treatment with acamprosate, disulfiram, or naltrexone. The treatment lasts for 3 months, and it is conducted by therapists, nurses, and social workers. Psychiatrists monitor the progression of patients during the treatment. For the treatment to be attuned to the individual patient, both the therapist and patient co-plan the course of the treatment, but typically the patient receives psychoeducation, is instructed in adaptive coping strategies (e.g., thinking about positive aspects of sobriety and negative aspects of drinking), and functional analyses are conducted for drinking scenarios.

### Phase 1–Parallel Randomized Controlled Trial

#### Recruitment

Patients from the alcohol outpatient clinic will be invited to participate on a consecutive basis until a total of 60 patients have been recruited. The clinic receives 600 patients over the course of 1 year.

#### Eligibility Criteria

To be eligible for participation in the pilot and feasibility study, patients must: (1) have a confirmed AUD diagnosis; (2) agree to participate in the study and provide verbal and written informed consent; (3) be aged between 18 and 60 years; (4) speak Danish; (5) have completed detoxification (if needed); (6) not have any sensory or motor deficits complicating the provision of the intervention (e.g., color-blindness, fine or gross motor deficits in upper extremities); (7) not meet diagnostic criteria for other substance use disorders (SUD); (8) not have a severe psychiatric or neurological illness (e.g., psychotic disorders, intellectual disability, or dementia) or terminal somatic illness; (9) own or be able to acquire a smartphone or tablet with internet access.

#### Enrollment and Randomization

Shortly after completing a personalized, pharmacological detoxification program (i.e., 1–2 weeks) at the outpatient facility and prior to starting primary treatment, the patients will be briefly informed of the study by the health care providers at the outpatient facility and asked if they would be willing to meet with a research assistant from the project for provision of full information on the study. The patients will also be informed about the possibility of bringing a visitor, either a friend, family member, or therapist at the clinic, for the meeting with the research assistant.

Before recruiting the patient, the research assistant will read out a standardized manuscript with information regarding the study, and the patients will also be provided with written information about the study. After this initial introduction, the research assistant will ask the patients whether they would be interested in participating in a baseline interview earliest the next day.

Upon receiving informed consent from the patients, the baseline interview will be administered, where the history of the AUD will be assessed (e.g., age of onset, life-time use, alcohol use, and alcohol excessive use). The first 60 patients fulfilling the eligibility criteria will shortly after the baseline interview be randomized to one of two groups. The intervention group (*n* = 30) will receive smartphone-based, multi-domain cognitive training with gamified elements together with treatment as usual (TAU), and the active control group (*n* = 30) will receive a sham version of the same cognitive training together with TAU.

#### Randomization Procedure

An urn randomization technique will be used to reduce bias and achieve balance in the allocation of patients to the intervention and active control groups. An allocation sequence will be provided by an off-site data manager (independent of the research team) and will be based on a computer-generated list of random numbers.

#### Intervention: Smartphone-Based, Multi-Domain Cognitive Training With Gamified Elements

The intervention consists of using Brain+ Recover, a gamified smartphone application developed by Brain+ ApS, which is available to the public and can be downloaded to iOS and Android devices. In the application, the patient has access to various cognitive training games, each targeting a set of complementary cognitive functions (see Appendix). The cognitive functions have been categorized by the developers into the following domains: attention (i.e., general visual attention and short-term memory), logic (i.e., planning, reasoning, and problem solving), perception (i.e., visual perception and WM), and memory (i.e., memory capacity). Each time the patient completes a game, feedback about performance is provided, and the level of difficulty is attuned accordingly (e.g., increment or decrement of speed, and higher or lower number of symbols, distractors, and obstacles). The first time the application is opened, the patient must complete a cognitive assessment, which is used to automatically create a personalized training program targeting the specific cognitive domains found to be impaired. Information about improvement in performance in each cognitive domain as well as a general cognitive profile can be accessed in the application itself at any given time. The application can either be used with Danish or English language, and this is automatically adapted to the default language on the device of the patient.

#### Active Control Group

The active control group will be provided with the same Brain+ Recover application as the experimental group. However, the difficulty of the tasks will be kept constant at a low level. Because of the fixed level of difficulty, the active control group will not have access to a brain profile. The active control group will receive the same instructions as the intervention group. This type of sham-cognitive training has been used in previous studies evaluating WM-specific serious gaming, which found no differences between the experimental and active control groups in terms of study completers (26, 27).

#### Usage of the Brain+ Recover Application

All patients will be helped with downloading the Brain+ Recover application on their own smartphone or tablet. All the games can be accessed at any time during the entire study period. The patients will be recommended to use the application for at least 20 min a day, 5 days a week, for 1 month (i.e., 20 sessions with 400 min of total training). This recommendation is based on previous studies on gamified cognitive training reporting improvements in cognitive outcomes after 2–5 sessions per week (each session varying between 45 and 60 min) with a total of 4–5 weeks of training [for more information, see (21–23)]. Actual time spent playing the games will be monitored. The patients are encouraged to allow notifications from the Brain+ Recover on their device, which will remind them about their daily training schedule once per day (e.g., Keep up the good work, remember to complete your daily training). There is, however, no option for the researchers to monitor whether the patients choose to deactivate the notification system after they leave the treatment facility.

#### Outcome Measures

The baseline interview will yield data on sociodemographic characteristics and AUD diagnosis and severity of the AUD (e.g., the age of onset, duration, alcohol consumption, and number of heavy drinking days). Cognitive outcomes will be assessed *via* the Brain+ Recover application at baseline and post-treatment. Clinical outcome measures will be assessed at baseline, post-treatment, and at 6-months follow-up.

The Mini-International Neuropsychiatric Interview (MINI) is a structured interview and will be used to confirm the diagnosis of AUD according to the criteria from the fifth version of the Diagnostic and Statistical Manual of Mental Disorders [DSM-5; (31)]. The cognitive outcomes include the cognitive functions trained by the Brain+ Recover application: processing speed and attention, executive functions, WM, and learning and episodic memory.

The Addiction Severity Index (ASI), a standardized international assessment instrument, will be used to generate an addiction severity profile for each patient. The profile covers seven areas of the patient's life: medical status, employment, drug use, alcohol use, legal status, family/social status, and psychiatric status (32). Based on the ASI alcohol concern area, ASI drinking measures will be derived from question A (days with any alcohol consumption in the past 30 days) and question B (days with excessive drinking, i.e., three units or more, in the past 30 days) hence reflecting the frequency and intensity of drinking, respectively. A composite ASI score will be calculated for each of the seven areas. The composite scores fall between 0 and 1, where 0 denotes no problems, 1 signifies severe problems (33). The primary outcomes are cognitive measures derived from Brain+ Recover, and the secondary outcomes are drinking measures and composite ASI scores for the seven problem areas.

#### Statistical Analysis

Repeated-measures Analysis of Variance (ANOVA), with group as the between-subjects factor and time of assessment as the within-subjects factor, will be used to test the effectiveness of the intervention across the assessment points, which will be indicated by a significant Time x Study Group interaction. If there is an overall significant interaction effect between any time-point and group, contrasts will be used to examine whether the change over time differs between the groups and the time-point. If the assumptions for the repeated measures ANOVA are violated, then a non-parametric equivalent will be used to address this issue. An intention-to-treat analysis and a completer analysis (on-training analyses) will be conducted for each outcome. The intention-to-treat analyses will be carried out for all patients, irrespective of whether they have completed the training or were re-interviewed. The significance level in the models will be set at α = 0.05, and two-tailed tests will be conducted. Effect sizes will be reported in accordance with the statistical modeling. All data will be analyzed in Stata version 16.

#### Power Analysis

Since this is a pilot study, no power calculation has been conducted. Nonetheless, prior small-scale studies have been able to detect effects on cognitive and clinical outcomes in similar sample sizes (include references).

### Phase 2–Feasibility Study

The 30 patients randomized to the intervention group will be invited to participate in the feasibility study. After completing the training, the patients will be asked to complete a short questionnaire focusing on their general experience with using the application (e.g., would you use it again?), which elements of the cognitive games they found the most engaging (e.g., processing speed, memory etc.), how they think the application can be improved (e.g., by adding daily training notifications, more feedback etc.), and what could motivate them to use the application more (e.g., increased therapist involvement, completing the training at the outpatient clinic). In addition, the validated System Usability Scale (SUS) will be used to evaluate how the user-interface is perceived by the patients (34).

Furthermore, three focus group interviews will be performed, for which 30 patients will be invited to participate. The themes of the focus group interviews will be inspired by the data recorded in the application and the questionnaires, and in particular address (1) patients' experience of the training; (2) possible improvements; and (3) aspects of importance when implementing the cognitive training as an adjunct to conventional AUD treatment. Furthermore, the patients that did not follow their daily training schedule or who refrained from using the application, will be asked whether they experienced any technical difficulties over the course of the study. Based on the feedback provided by the patients, consisting of both quantitative and qualitative data, the Brain+ Recover application will be modified and subsequently renamed Brain+ AlcoRecover before implementing it in the subsequent large-scale RCT (expected *n* = 252).

To progress to the large-scale RCT, the patients must have used the application at least eight times for a total of 80 min during the entire study period (i.e., 20% of the recommended usage time). If this is not achieved in the first trial, a further 60 patients will be recruited and invited to participate in another pilot study using an adapted version of Brain+ AlcoRecover.

### Data Management

The data collected during the baseline interview and during the 6-month follow up will be treated as strictly confidential and managed by Odense Patient Data Explorative Network (OPEN). After the patients have been randomized to either of the two groups, they are assigned an ID-number, which ensures that the data collected through the Brain+ Recover application is anonymous so that the data cannot be traced back to any of the personal information of the patient. Treatment of data will comply with the Data Protection Regulation and the Data Protection Act. No analysis or publication will contain information that allows person identification.

### Economical Compensation

Since this is a study examining a psychological intervention expected to cause no damage and, in the worst-case scenario, only cause transient and minor discomfort, there is no economical compensation system linked to the trial.

### Economy

The study was developed on the initiative of the Unit of Clinical Alcohol Research and is unconditionally funded by the Psychiatric Research Foundation, Region of Southern Denmark.

### Ethical Considerations

All the patients in this study will be offered either pharmacological or psychological treatment at the outpatient alcohol clinic. There are no known harmful effects of the smartphone-based training program. Hence, the current study does not pose any ethical problems, and it will adhere to the World Medical Association Declaration of Helsinki. In addition, the protocol has been approved by the Research Ethics Committee for Southern Denmark (Project ID: S-20200199) and will be conducted in accordance with the General Data Protection Regulation. Before signing the consent agreement for participation in the research project, the patients are informed that they at any given time during the study have the option to withdraw their consent without it having any implications for their current or future treatment options. If the patients choose to withdraw their consent, they are informed that all their data will be erased.

### Perspectives

Since the present study relies on feedback regarding modifications to the Brain+ Recover application, patients with AUD will take active part in the development of the final version of Brain+ AlcoRecover. Collaboration with the patients will ensure that the smartphone-based cognitive training is well-suited to the requirements of patients with AUD. Thus, this study will uncover how compliance and adherence to gamified and smartphone-based cognitive training can be optimized for it to be a putative instrument for clinical practice in terms of the future treatment for AUD. In keeping with this, to bridge the gap between research and clinical practice even further and to maximize the effectiveness of the intervention, the current study will also include patients who underwent pharmacological detoxification recently (e.g., 1 day after completing detoxification program) well-knowing that such treatment with benzodiazepines have an impact on cognitive functions [for more information, see (35, 36)].

In this study the mHealth-based cognitive training is delivered as adjunctive treatment, therefore, it is hypothesized that it will be less disruptive for the conventional treatment, which in turn would make it easier to adapt it to the clinical facilities. If the Brain+ Recover shows a trend toward effectiveness and feasibility, this pilot study will constitute the fundament for a future large-scale RCT in which the effects of smartphone-based, multi-domain cognitive training with gamified elements delivered as an adjunct to TAU will be compared to sham-control training in combination with TAU as well as TAU only. Given the scarcity of evidence on SG and cognitive training as adjunctive treatment delivered through smartphone applications, which could create the groundwork for future research to explore the effects of mHealth-based cognitive training in patients with AUD. The cognitive heterogeneity of patients with AUD emphasizes the need of this type of mHealth focused research, as it could create the groundwork for more motivating personalized treatment options. Future smartphone based personalized cognitive training could also give patients the opportunity to be more in charge of their own treatment, making it more anonymous and less stigmatizing for the patients.

## Data Availability Statement

The original contributions presented in the study are included in the article/Supplementary Material, further inquiries can be directed to the corresponding author.

## Ethics Statement

The studies involving human participants were reviewed and approved by the Research Ethics Committee for Southern Denmark (Project ID: S-20200199) and will be conducted in accordance with the General Data Protection Regulation. The patients/participants provided their written informed consent to participate in this study.

## Author Contributions

AM and NM wrote the manuscript. All authors were responsible for the design of the whole study, wrote the protocol, supported the manuscript preparation, and approved the final manuscript.

## Conflict of Interest

The authors declare that the research was conducted in the absence of any commercial or financial relationships that could be construed as a potential conflict of interest.

## Publisher's Note

All claims expressed in this article are solely those of the authors and do not necessarily represent those of their affiliated organizations, or those of the publisher, the editors and the reviewers. Any product that may be evaluated in this article, or claim that may be made by its manufacturer, is not guaranteed or endorsed by the publisher.
