# Supplementary material for: Brain+ AlcoRecover: A Randomized Controlled Pilot-Study and Feasibility Study of Multiple-Domain Cognitive Training Using a Serious Gaming App for Treating Alcohol Use Disorders
Source: Front Psychiatry. 2021 Oct 1;12:727001. doi: 10.3389/fpsyt.2021.727001 (PMC8517229; doi:10.3389/fpsyt.2021.727001)
Supplement: Supplementary file 1 [file Data_Sheet_1.pdf]

Appendix

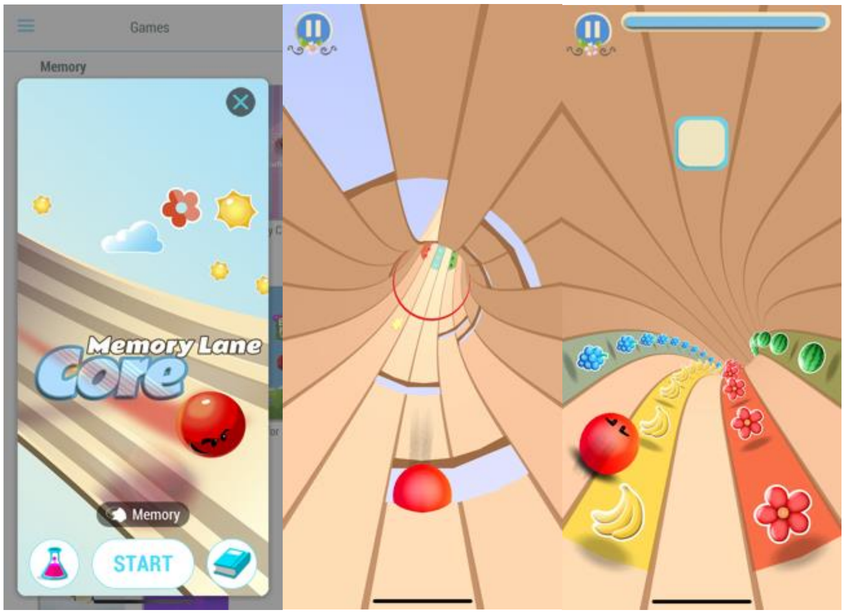

Figure 1. Sample of the game "Memory Lane" that trains attention and short-term visual memory. The task consists of remembering and collecting the right symbols (e.g., banana, flower, watermelon) in the correct sequence.

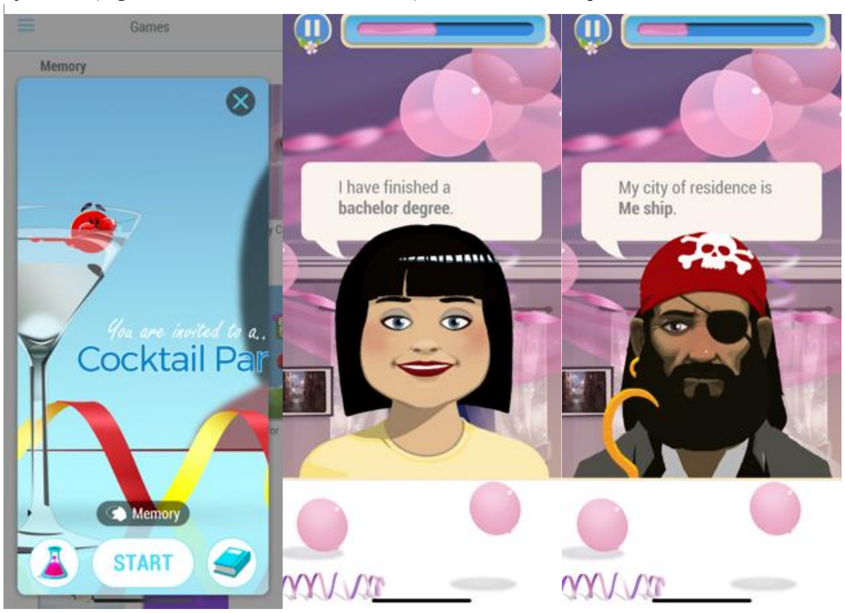

Figure 2. A sample of the game "Attention Island" that trains visual attention and short-term memory. Here the individuals are presented for white shells that are placed in various places for a brief time. The task is to guide the red character to the designated area, and then selecting the placement of the white shells.

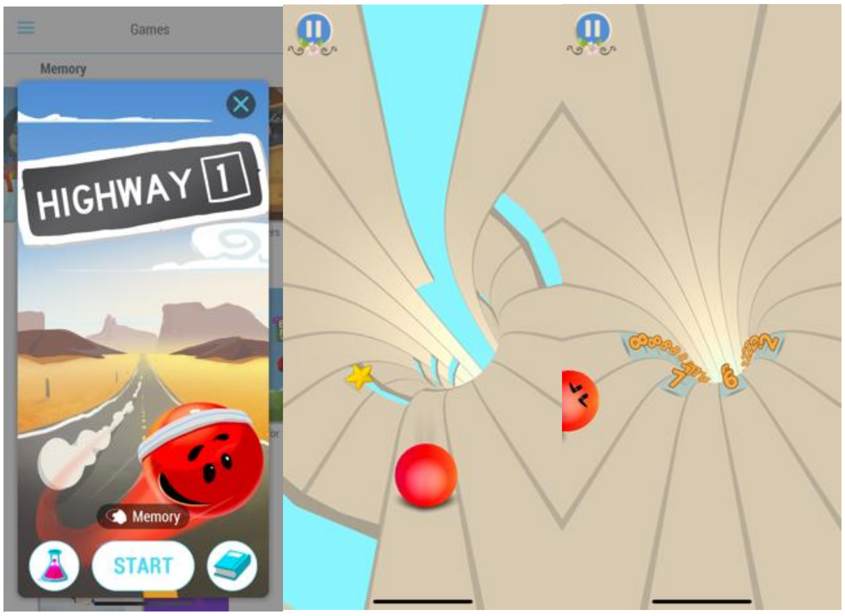

Figure 3. A sample of the game “Highway 1” that trains visual attention and short-term memory specifically for digits. Here the individuals must remember the correct digits and sequence presented along the highway.

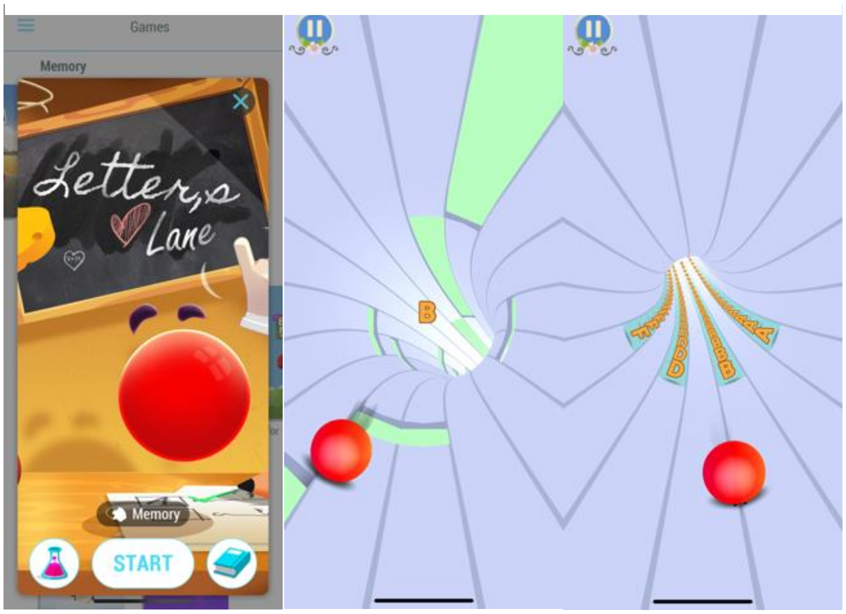

Figure 4. A sample of the game “Letter’s Lane” that trains visual attention and short-term memory specifically for letters. Here the individuals are presented with different letters that they must remember in the right sequence.

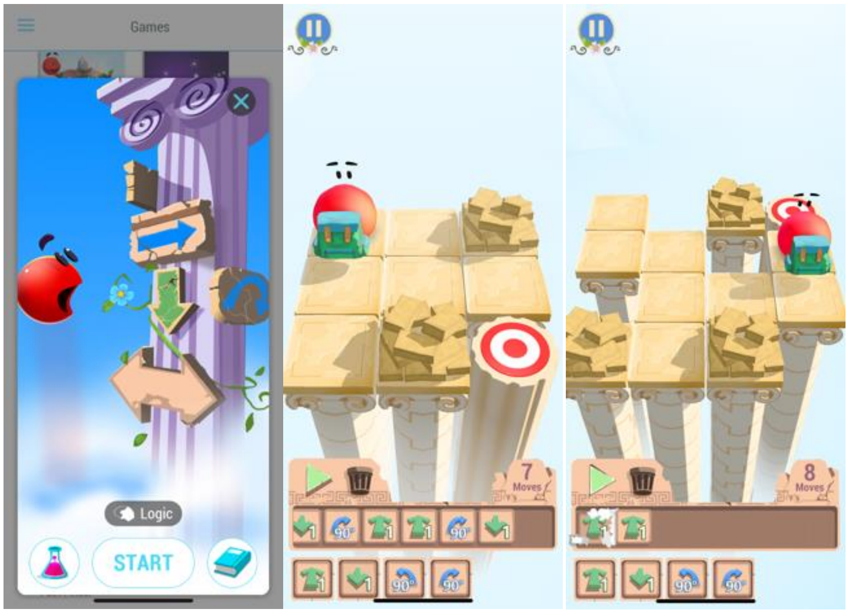

Figure 5. A sample of the game “Path Finder” that trains problem solving abilities. Here the task is to plan the right path for the red character, for it to reach the red target.

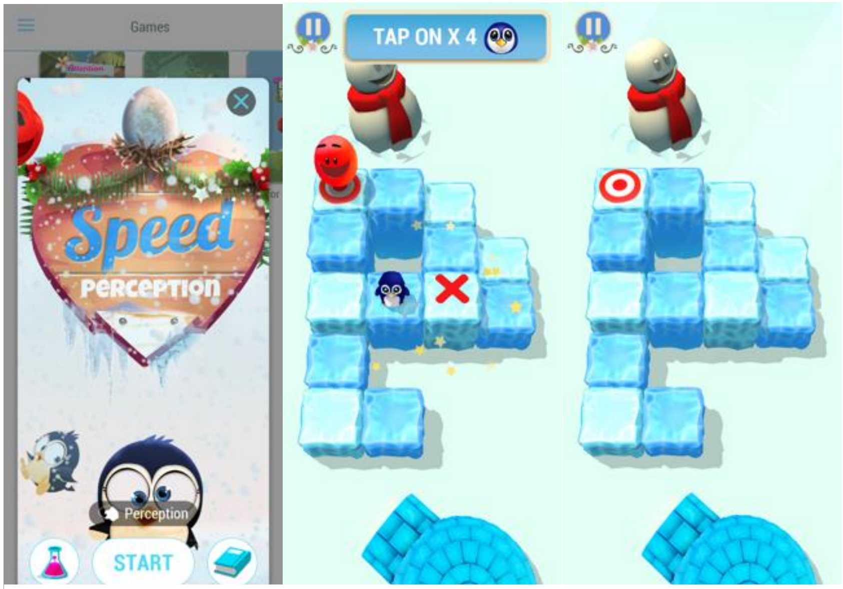

Figure 6. A sample of the game “Speed Perception” that trains visual attention and short-term memory. Here the task is to remember the placement of the penguins that are only presented for a brief time. The individual must guide the red character to the targeted area and selecting the correct placement of the penguins.

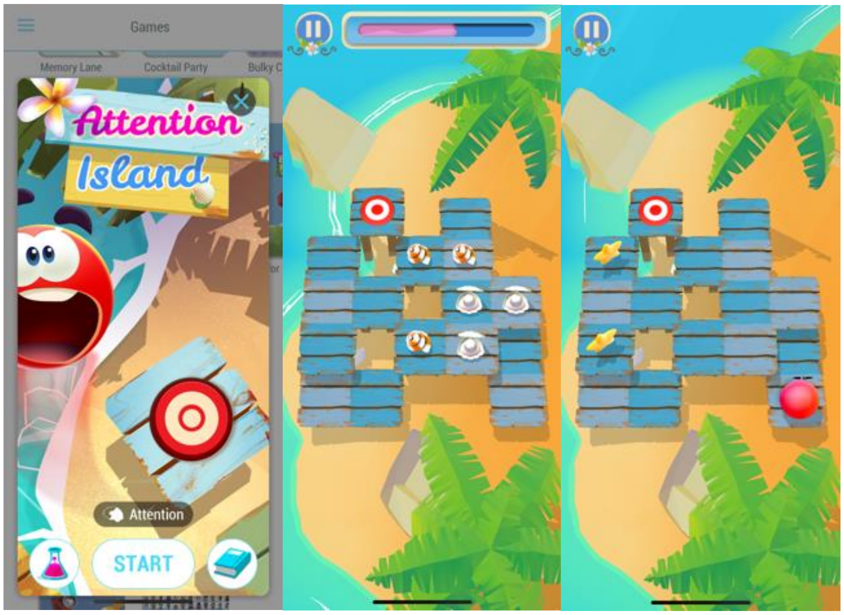

Figure 7. A sample of the game “Attention Island” that trains visual attention and short-term memory. Here the individuals are presented for white shells that are placed in various places for a brief time. The task is to guide the red character to the designated area, and then selecting the placement of the white shells.

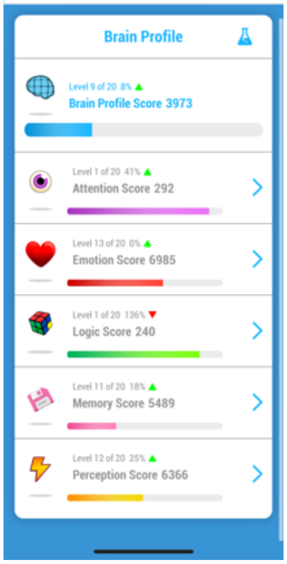

Figure 8. A screenshot of the page for the “Brain Profile”, which the patients in the experimental group will have access to at any given time. Here they can track their progress in various cognitive domains (i.e. levels from 1 to 20).
